# Sonochemical synthesis of SnS and SnS_2_ quantum dots from aqueous solutions, and their photo- and sonocatalytic activity

**DOI:** 10.1016/j.ultsonch.2024.106834

**Published:** 2024-03-06

**Authors:** Grzegorz Matyszczak, Tomasz Plocinski, Piotr Dluzewski, Aleksandra Fidler, Cezariusz Jastrzebski, Krystyna Lawniczak-Jablonska, Aleksandra Drzewiecka-Antonik, Anna Wolska, Krzysztof Krawczyk

**Affiliations:** a Department of Chemical Technology, Faculty of Chemistry, Warsaw University of Technology, Noakowski street 3, 00-664 Warsaw, Poland; b Faculty of Materials Science and Engineering, Warsaw University of Technology, Wołoska street 141A, 02-507 Warsaw, Poland; c Institute of Physics Polish Academy of Sciences, Poland, Lotników avenue 32/46, 02-668 Warsaw, Poland; d Faculty of Physics, Warsaw University of Technology, Koszykowa street 75, 00-662 Warsaw, Poland

**Keywords:** Tin(IV) sulphide, tin(II) sulphide, Nanoparticles, Sonochemistry, Sonocatalysis, Photocatalysis

## Abstract

Our study reports the ultrasound-assisted synthesis of SnS and SnS_2_ in the form of nanoparticles using aqueous solutions of respective tin chloride and thioacetamide varying sonication time. The presence of both compounds is confirmed by powder X-ray diffraction, energy-dispersive X-ray spectroscopy, as well as Raman and FT-IR spectroscopic techniques. The existence of nanoparticles is proven by powder X-ray diffraction investigation and by high resolution transmission electron microscopy observations. The size of nanocrystallites are in the range of 3–8 nm and 30 50 nm for SnS, and 1.5–10 nm for SnS_2_. X-ray photoelectron spectroscopy measurements, used to investigate the chemical state of tin and sulphur atoms on the surface of nanoparticles, reveal that they are typically covered with tin on the same oxidation degree as respective bulk compound. Values of optical bandgaps of synthesized nanoparticles, according to the Tauc method, were 2.31, 1.47 and 1.05 eV for SnS (60, 90 and 120 min long synthesis, respectively), and 2.81, 2.78 and 2.70 eV for SnS_2_ (60, 90 and 120 min long synthesis, respectively). Obtained nanoparticles were utilized as photo- and sonocatalysts in the process of degradation of model azo-dye molecules by UV-C light or ultrasound. Quantum dots of SnS_2_ obtained under sonication lasting 120 min were the best photocatalyst (66.9 % color removal), while quantum dots of SnS obtained under similar sonication time were the best sonocatalyst (85.2 % color removal).

## Highlights

• SnS and SnS_2_ are synthesized in the form of quantum dots from aqueous solutions.
• Syntheses are conducted under room conditions thanks to application of ultrasound.
• Influence of sonication time on QDs properties is investigated.
• SnS and SnS_2_ QDs were utilized as photo- and (for the first time) sonocatalysts.
• The greatest color removal was observed in the case of SnS QDs sonocatalyst (85.2%)

## Graphical abstract

**Figure:**
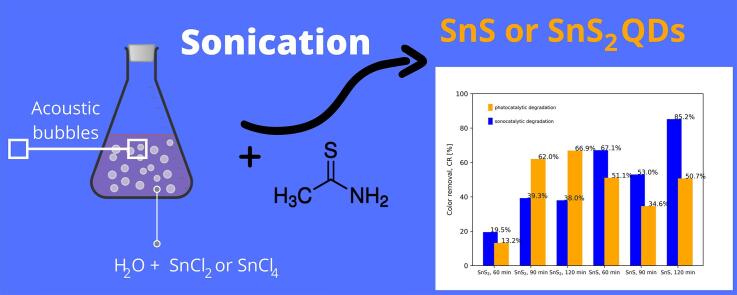

## 1 Introduction

Sonochemistry is a division of chemistry which studies the overall chemical effects of interaction of ultrasound with investigated system. During the passing of ultrasound wave through liquid a fluctuations of pressure are created what affects the solubility of gases in liquid. This leads to formation of cavitation bubbles which grow through the diffusion into their volume of gases dissolved in liquid. When a bubble reaches its maximum size it collapses generating relatively very high temperatures and pressures of orders of magnitudes 10^4^ K and 10^3^ atm respectively [1], [2]. Under such extreme conditions many chemical reactions may occure. One such example is the sonolysis of water forming radicals H**^·^** and **^·^**OH, which is probably the most basic sonochemical reaction [1], [2]:

H_2_O → H**^·^** + **^·^**OH

Many applications of sonochemistry include synthesis of inorganic materials, which composition may vary from simple (two-element, e.g. ZnS, Bi_2_S_3_) to complex (many-element, e.g. CuInS_2_, Cu_3_BiS_3_, Cu_2_ZnSnS_4_) [3], [4], [5], [6], [7]. They may be obtained in forms varying from nano- to microparticles of diverse morphology. The control of size and shape of synthesized materials may be further improved by merging sono- and electrochemical syntheses [8], [9], [10], [11]. The most important thing about the sonochemical synthesis is that it is typically conducted at mild conditions (room temperature, normal pressure) and in many cases it allows using water as a solvent thus avoiding usage of toxic and hazardous organic solvents used in methods such as solvothermal synthesis or hot-injection reaction [2], [3], [4]. Moreover, the application of ultrasound increases the rate of reaction without the addition of catalyst [2]. Overall, the sonochemical processes meet the criteria of so-called “green chemistry” and have great potential for future applications, especially in environment friendly industrial processes.

Among inorganic materials that may be obtained via sonochemical route tin sulphides - SnS and SnS_2_ - may be distinguished. Both of them are very important and are widely investigated in terms of their applications. They include optoelectronics, photovoltaics, photocatalysis, photonics and many more [12], [13], [14], [15], [16]. Chemical methods of preparation of SnS or SnS_2_ in the form of nanostructures include solvothermal reaction, hot-injection reaction, polyol method, and precipitation from aqueous solutions [17], [18], [19], [20], [21], [22], [23], [24]. Sonochemical synthesis was also applied for production of tin sulphides. SnS and SnS_2_ were prepared in that way in many forms and with varying properties such as optical energy bandgap using distinct reagents and solvents [25], [26], [27], [28], [29], [30]. A systematic study of influence of various synthetic conditions such as solvent (ethanol or ethylenediamine), sonication time, proportions of reagents, and tin source (SnCl_2_ or SnCl_4_) in the sonochemical synthesis of tin sulphides was conducted revealing that properties of product such as optical energy bandgap, mean particle size, morphology of particles and phase composition soundly depends on synthetic conditions [31]. Tin sulphides are perpetually investigated in the wide spectrum of applications, which include: electrocatalysis, gas sensors, humidity sensing, energy storage, and Li-ion batteries, solid-state extraction of antibiotics, photodetectors, and photocatalysis [32], [33], [34], [35], [36], [37], [38], [39]. However, a method of obtaining of SnS and SnS_2_ quantum dots from aqueous solutions under ultrasound radiation wasn’t reported so far. This is the main novelty of the presented study.

The synthesized quantum dots were then utilized as catalysts in the processes of degradation of model organic pollutant. The development of civilization is inevitably related with increasing pollution of natural environment. This may cause irreversible damage to the nature and also affect human health. For example, in the Ganges River Basin in India in surface- and groundwater which is used without purification as drinking water by people, occurrence of at least dozen of biologically active compounds (e.g. caffeine, acetaminophen, diclofenac and ibuprofen) was confirmed [40], [41]. On the other hand, in Europe, Catalonia (north-east region of Spain) a 4-year monitoring of groundwater proved that concentration of 13 different pesticides was above the limit set by European Commission [42]. Many attention is paid for development of methods of removal of organic pollutants from waters. Among them a photocatalytic and sonocatalytic processes may be distinguished [43], [44]. For example, WS_2_@CeO_2_ heterostructure was used in the sonocatalytic degradation of tylosin (a macrolide antibiotic), while composites CeO_2_/TiO_2_, SnO_2_/TiO_2_ and ZrO_2_/TiO_2_ were used as sonocatalysts for degradation of organic dye Acid Red B [45], [46]. However, despite many investigated sonocatalysts and many applications of tin sulphides, up to this time SnS and SnS_2_ weren’t utilized as sonocatalysts. This is another novelty of our study.

This study presents the sonochemical synthesis of quantum dots of SnS and SnS_2_ from aqueous solutions. Products of syntheses are well-characterized by following techniques: X-ray powder diffraction (PXRD), scanning electron microscopy (SEM) coupled with energy dispersive spectroscopy (EDS), Fourier transform infrared spectroscopy (FT-IR), Raman spectroscopy, high-resolution transmission electron microscopy (HR-TEM), UV–Vis spectrophotometry (including determination of optical bandgap by Tauc method), and X-ray photoelectron spectroscopy (XPS). The properties of obtained SnS and SnS_2_ are thus determined and compared. Synthesized quantum dots were also utilized as photo- and sonocatalysts in the process of removal of model azo-dye (Metanil Yellow) from aqueous solutions, and their activity is compared.

## 2 Material and methods

### 2.1 1. Materials and reagents

All chemicals used in this study were pure for analysis (producer: POCH). For sonochemical syntheses, SnCl_4_·5H_2_O, SnCl_2_·2H_2_O and thioacetamide (TAA) were used as reagents. Distilled water was used as a solvent and ethanol was used for the purification of prepared suspensions.

### 2.2 Sonochemical syntheses and purification of products

The sonochemical syntheses were conducted in conical flasks of 50 mL volume in an ultrasonic cleaner (PS 10A) generating an ultrasound of 40 kHz frequency with nominal power of ultrasounds 60 W. The acoustic power of utilized cleaner was 27.9 W/L according to calorimetric measurements. The procedure for syntheses and purification of obtained powders was adopted from previous Matyszczak et al. investigation [31].

In all syntheses following amounts of reagents were used: 701 mg of SnCl_4_·5H_2_O or 452 mg of SnCl_2_·2H_2_O, 376 mg of thioacetamide, and 20 mL of distilled water as solvent. All reagents were stirred for 10 min with a magnetic stirrer before the start of the reaction. The duration of sonication was 60 and 120 min. After that and before the purification the open conical flasks with obtained powders suspended in original reaction mixture were kept under laboratory hood for 10 min to remove the toxic gases generated during reaction.

The products of syntheses were separated from the reaction mixture by centrifugation. After first centrifugation, a supernatant (reaction mixture) was removed and portion of fresh ethanol was added. Then the powder was suspended in the fresh ethanol by sonication (10 min) and mixing by plastic Pasteur pipette. Then the centrifugation was repeated, supernatant removed, and another portion of fresh ethanol was added. After that, powder was again suspended in fresh ethanol. Then the suspension was centrifuged, supernatant was removed and the powder was finally suspended in another portion of fresh ethanol.

### 2.3 Evaluation of sonochemical efficiency and percentage yield of the synthesis process

The percentage yield and the sonochemical efficiency of the synthesis process were calculated according to the two following equations:

$$Y_{t}=\frac{m_{t,dry}}{m_{\mathrm{theoretical}}}\cdot 100\text{\textbackslash{}\%}$$

$${\mathrm{SE}}_{t}=\frac{m_{t,dry}}{P_{\mathrm{ultrasound}}}$$

where:

$Y_{t}$- percentage yield of sonochemical reaction of duration time t [%]
$m_{t,dry}$- mass of dried product of reaction lasting time t [µg]
$m_{\mathrm{theoretical}}$- theoretical mass of dried product calculated based on taken amount of reagents assuming full (100 %) overreaction [µg]
${\mathrm{SE}}_{t}$- sonochemical efficiency of reaction lasting time t [µg/W]
$P_{\mathrm{ultrasound}}$- power of ultrasound determined calorimetrically [W].

Both values (Y and SE) are calculated in reference to the dried products which were obtained by drying of purified suspensions in ethanol under laboratory hood for approximately 3 days.

### 2.4 Uv–vis spectrophotometry (Tauc method)

The UV–Vis spectrum of obtained suspension of product of synthesis in ethanol was collected using UV1600 spectrophotometer (AOE Instruments) and then was used to perform analysis based on the Tauc method:

$${\left(\alpha h\nu \right)}^{\frac{1}{n}}=A\left(h\nu -E_{g}\right)$$

where:

α – the linear absorption coefficient (cm^−1^)
hν – the energy of electromagnetic wave (eV)
A – constant
E_g_ – the optical energy bandgap (eV)
n – the number associated with distinct electron transition type: n = $\frac{1}{2}$ for direct allowed transition, n = $\frac{3}{2}$ for direct forbidden transition, n = 2 for indirect allowed transition, n = 3 for indirect forbidden transition.

### 2.5 FT-IR investigation

The Fourier-transform infrared spectroscopy measurements were conducted using NICOLET 6700 FT-IR spectrometer. Dried powders of synthesized materials were grounded with KBr and formed as pellets.

### 2.6 Raman spectroscopy investigation

Micro-Raman measurements were conducted using a Renishaw inVia Reflex spectrometer in backscattering geometry. The 633 nm line of the He-Ne–ion laser was used as the excitation. Raman spectra were collected at room temperature and under normal conditions. The spectral resolution of the measured spectra in this configuration was about 1.2 cm^−1^.

### 2.7 XPS investigation

The suspensions of samples were dripped on a conductive tape and left to dry in laboratory fume cupboard. The procedure was repeated until a visible sample layer was obtained then samples were introduced to the spectrometer. X-ray photoelectron spectra (XPS) were recorded by Prevac set-up equipped with high intensity monochromatic X-ray Al Kα (1486.69 eV) source Scienta MX 650 (set at 300 W), Scienta R4000 hemispherical analyser, and charge neutralization. The wide spectra were registered with pass energy 500 eV and the step 0.5 eV. The narrow scans were acquired with pass energy 200 eV and the step 0.2 eV for Sn 3d line and step 0.1 for S 2p line. The full width at half maximum (FWHM) for the Au 4f 7/2 line measured at the same experimental condition was equal to 0.6 eV regardless of energy step. The energy scale was calibrated setting the C 1 s line at the position 284.5 eV. Spectra were analysed using the commercial CASA XPS software package (Casa Software Ltd, version 2.3.17) [47] with Shirley background and a GL(30) line shape (70 % Gaussian, 30 % Lorentzian).

### 2.8 SEM investigations

The Scanning Electron Microscopy SEM investigations were performed on the microscope made by Hitachi model SU8000. The powder samples were deposited on carbon tape after drying. The observations were taken at low voltage range between 3 up to 5 kV accelerating voltage. The Secondary Electrons SE were used to register images of surface topography at magnification range of 40 000 times.

### 2.9 Powder X-ray diffraction

The powder diffraction data were collected for dried samples using X-ray diffractometer Bruker D8 ADVANCE and filtered Cu Kα radiation (λ = 0.154056 nm) at room temperature. The parameters during measurements were as follows: voltage − 40 kV, current – 40 mA, angle range from 10° to 80°, step Δ2Θ − 0.025°, counting time – 3 s.

### 2.10 HR-TEM and EDX investigations

One drop of the powder suspended in ethanol was deposited on a standard TEM copper grid coated with an amorphous carbon film. After the grid went completely dry it was ready to put into the microscope.

Investigations were conducted on the FEI Titan Cubed 80–300 TEM with an accelerating voltage of 300 kV and the point resolution of 130 pm. The overview images were registered in bright-field TEM mode for magnifications ranging from 9.800x to 55.000x. For the purpose of high-resolution imaging magnifications from 145.000x − 380.000x range were used. In both cases, the images were obtained with the Gatan BM-Ultrascan CCD camera. STEM (scanning mode) images were acquired with the use of the HAADF detector which registers strongly scattered electrons. The in situ EDAX spectrometer was used for the analysis of the specimen's elemental composition.

### 2.11 Photo- and sonocatalytic experiments

**Fig. 1:**
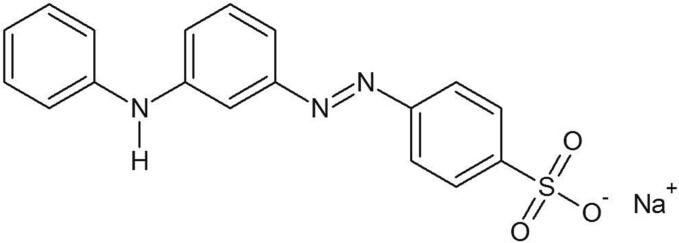
Structural formula of azo-dye Metanil Yellow.

For degradation experiments we have chosen azo-dye Metanil Yellow which structural formula is displayed in Fig. 1. Despite its high toxicity it is illegally used in food industry especially in India, e.g. as adulterant in turmeric powders [48], [49]. There are evidences for mutagenic and carcinogenic actions of azo-dyes against human [50], [51], [52].

Before both photo- and sonocatalytic experiments a 30 mL of dye solution in distilled water was placed in the beaker equipped with magnetic bar. A portion (20 mg) of specific tin sulphide powder was weighed and placed in 5 mL of distilled water and sonicated for 40 min. Such a suspension was then added to the beforehand measured 30 mL of dye solution. To ensure the quantitative transfer of catalyst to the dye solution, the leftovers of catalysts suspension were washed 5 times with 1 mL of distilled water (each time additionally applying sonication for 10 s). Finally the volume of dye solution mixed with the suspension of catalyst was 40 mL. Each volume was measured using the graduated pipette. The prepared mixture was then stirred on magnetic stirrer with speed of 800 rpm under dark conditions for 30 min to ensure the adsorption–desorption equilibrium. After that, the mixture was used in sono- or photocatalytic experiments.

The photocatalytic process was conducted in a initially used glass beaker of 50 mL volume under ambient temperature and pressure. The source of UV radiation was the UV-C lamp (power 72 W). Each time the position of beaker in reference to the UV lamp, as well as the shape and size of beaker, was exactly the same. The UV irradiation lasted 120 min in each photocatalytic experiment. Experiments were conducted under dark conditions.

The sonocatalytic experiments were conducted in falcons (centrifuge tubes) of 14 mL volume. For one experiment a portion of 7 mL of prepared catalysts suspension in dye solution was measured with pipette and placed in each falcon. Four falcons were then placed in ultrasonic bath under dark conditions and sonicated for 120 min. The positions of falcons in ultrasonic bath were precisely controlled and kept the same in each experiment. The level of water in bath was equal to the level of liquid in falcons.

After both kind of processes the mixture was centrifuged (8000 rpm, 8 min) twice to obtain clear dye solution, free of suspended catalyst and ready for absorbance measurement. The absorbance was measured at 440 nm, corresponding to the maximum of absorbance of Metanil Yellow aqueous solution, using UV–Vis spectrophotometer. Each experiment, including blind test, was conducted 4 times.

The color removal during the processes was calculated according to the following straightforward equation:

$$\mathrm{CR}=\frac{A_{O}-A_{E}}{A_{O}}\cdot 100\%$$

Where:

CR- percentage color removal [%]
$A_{0}$- absorbance of dye solution before sono- or photocatalytic process
$A_{E}$- absorbance of dye solution after sono- or photocatalytic process.

## 3 Results and discussion.

### 3.1 Investigation of synthesis efficiency

Products of syntheses formed uniform suspensions of yellow-orange color (in the case of SnS_2_ syntheses) or dark-brown color (in the case of SnS syntheses). Suspensions obtained in the syntheses lasting for 120 min had more intensive colors compared to those obtained in 60 min long reactions, what is undoubtedly caused by formation of greater amount of product under longer sonication. The calculated percentage yields of performed sonochemical syntheses as well as sonochemical efficiencies of formation of SnS and SnS_2_ in varying time of synthesis are presented in Table 1. As may be seen, almost full overreaction of SnCl_4_ to SnS_2_ may be achieved under sonication lasting 120 min while at the same conditions SnCl_2_ is converted to SnS in less than 50 %. The calculated values of sonochemical efficiencies show that in reaction lasting 120 min it is possible to obtain c.a. 13 mg of SnS_2_ and c.a. 5 mg of SnS in reference to ultrasound of power 1 W. It may be concluded that the sonochemical synthesis of SnS_2_ is more efficient than sonochemical synthesis of SnS.

**Table 1:** Percentage yields and sonochemical efficiencies of performed syntheses.

Tin sulphide	Sonication time (min)	$Y_{t}$(%)	${\mathrm{SE}}_{t}$(mg/W)
SnS_2_	60	91.5	11.99
	90	98	12.87
	120	99	12.98
SnS	60	23	2.49
	90	35	3.82
	120	46	4.97

### 3.2 X-ray powder diffraction investigations

**Fig. 2:**
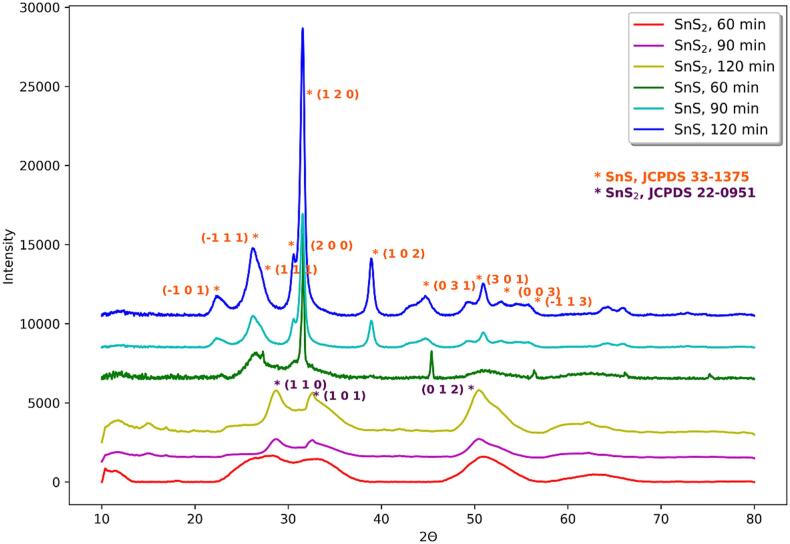
Powder X-ray diffractograms diffraction patterns of products of syntheses sonochemically synthesized SnS and SnS_2_ quantum dots.

The X-ray powder diffraction investigations confirmed the presence of both compounds, SnS and SnS_2_, in nanocrystalline forms as indicated by presence of wide reflexes in the diffraction patterns (Fig. 2). The absence of wide, large peak at the beginning of diffraction pattern suggests that prepared samples are rather nanocrystalline than nanoamorphous. Products of longer syntheses (120 or 90 min) have generally more and/or better shaped reflexes than products of syntheses lasting 60 min. This is apparently due to the greater amount of products after longer sonication. Also, in the case of SnS obtained after 90 and 120 min of sonication more reflexes are present what may be linked to better crystallinity of sample.

### 3.3 FT-IR investigations

The Fourier transform infrared spectroscopy investigation confirms the presence of tin sulphides in prepared samples. The corresponding spectra are presented in Figure S1. They show the occurrence of trace amounts of solvents used in syntheses (H_2_O) and purification (C_2_H_5_OH) as indicated by peaks corresponding to C-C and C-H bonds, and –OH groups. The bands related to Sn-S bond are similar in both samples of SnS_2_ what is in line with the XRD result. However, significant difference is observed between spectra of SnS obtained after 60 and 120 min of sonication. In the latter case the band is much more defined revealing that the product of longer synthesis is of better crystallinity. This result is also in line with the XRD result.

### 3.4 Raman spectroscopy investigations

**Fig. 3:**
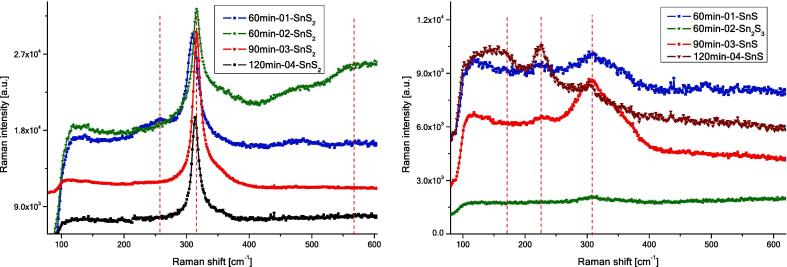
(Left) Typical Raman spectra of as-synthesized nano SnS_2_ samples. Vertical dashed lines were placed for 255 cm^−1^, 315 cm^−1^ and 570 cm^−1^. (Right) Typical Raman spectra of as-synthesized nano SnS samples. Vertical dashed lines were placed for 160 cm^−1^, 225 cm^−1^ and 310 cm^−1^.

The Raman studies were performed to investigate the microstructural phase homogeneity of the sulphide samples. The studies have shown that the investigated samples are homogeneous in terms of chemical composition. For products of sonochemical syntheses of SnS_2_ the recorded spectra corresponded to the rhombohedral structure of SnS_2_. This is evidenced by a clear peak of A_1g_ symmetry for about 315 cm^−1^ (Fig. 3, on the left) [53], [54]. In the case of SnS_2_ obtained under sonication lasting 60 min, for a few measurements, a slight shift of this peak towards lower frequencies was observed. This shows that we are dealing with different polytypes of the SnS_2_ structure [55]. Some asymmetry was also observed for the 315 cm^−1^ peak. As a rule, such asymmetry occurs when phonons interact with free carriers or is the result of phonon constraints in nanostructures [56], [57]. The low excitation energy used in the Raman studies (633 nm) in relation to the width of the SnS_2_ energy gap allows excluding the interference factor between phonons and electrons from the conduction band. The asymmetry of the peak should therefore be interpreted as an effect of the small size of SnS_2_ grains and the resulting phenomena of quantum confinement.

In the case of products of sonochemical syntheses of SnS, the spectrum corresponds to that recorded for SnS nano grains [53], [58]. Both peaks present in the spectra for about 225 cm^−1^ and about 305 cm^−1^ (Fig. 3, on the right) are rather present in poorly crystallized SnS nano grains. In the case of nanostructures with SnS stoichiometry, the literature data shows the presence of both peaks, for 225 cm^−1^ and for about 305 cm^−1^ [58]. The peak at 225 cm^−1^ comes from interatomic vibration between metal (Sn) and chalcogen (S). With the advancing crystallization process, the peak at about 190 cm^−1^ [53] becomes dominant for bulk samples of SnS, which was not observed in the tested samples. At the same time, in crystalline bulk samples of SnS, the peak for about 305 cm^−1^ disappears. The peak at about 305 cm^−1^ can also be interpreted as the presence of the Sn_2_S_3_ phase, especially since for some grains only a wide peak at 305 cm^−1^ was recorded (spectrum 120 min-02-Sn_2_S_3_, Fig. 3, right). For Sn_2_S_3_ structure the peak at 305 cm^−1^ is associated with the intralayer vibration of chalcogen–chalcogen ions [58].

### 3.5 Scanning electron microscopy investigations

**Fig. 4:**
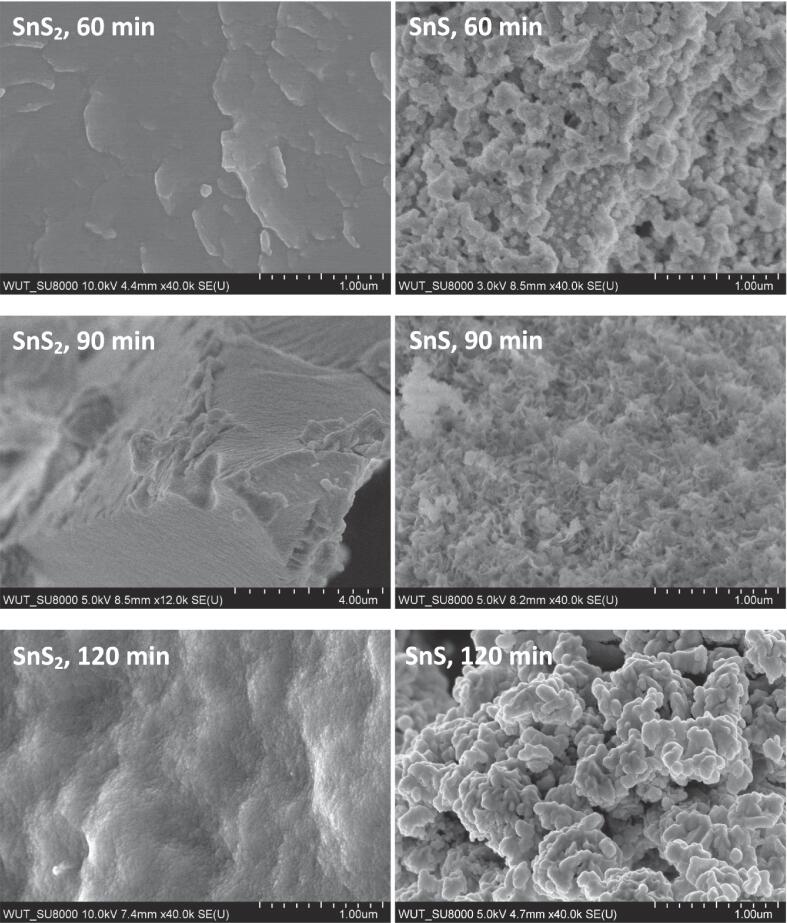
SEM images of sonochemically synthesized tin sulphides.

Scanning electron microscopy observations were used to study the morphology of synthesized tin sulphides (Fig. 4). The dried powder of SnS_2_ synthesized under sonication lasting 60 min shows a bit rough surface without any characteristic particles. In the case of SnS_2_ synthesis with duration of 90 and 120 min, the dried product had wavy surface, and very small particles with size smaller than 0.1 μm (100 nm) may be observed. SEM images of SnS powders reveal the presence of rather uniform particles of granular shape and submicron size. SnS powder synthesized with longer sonication time exhibited larger particles. EDX measurements confirmed the elemental composition of SnS and SnS_2_ samples. Relevant Sn:S atomic ratios are nearly 1:1 for SnS and 1:2 for SnS_2_ samples.

### 3.6 High-resolution transmission electron microscopy investigations

**Fig. 5:**
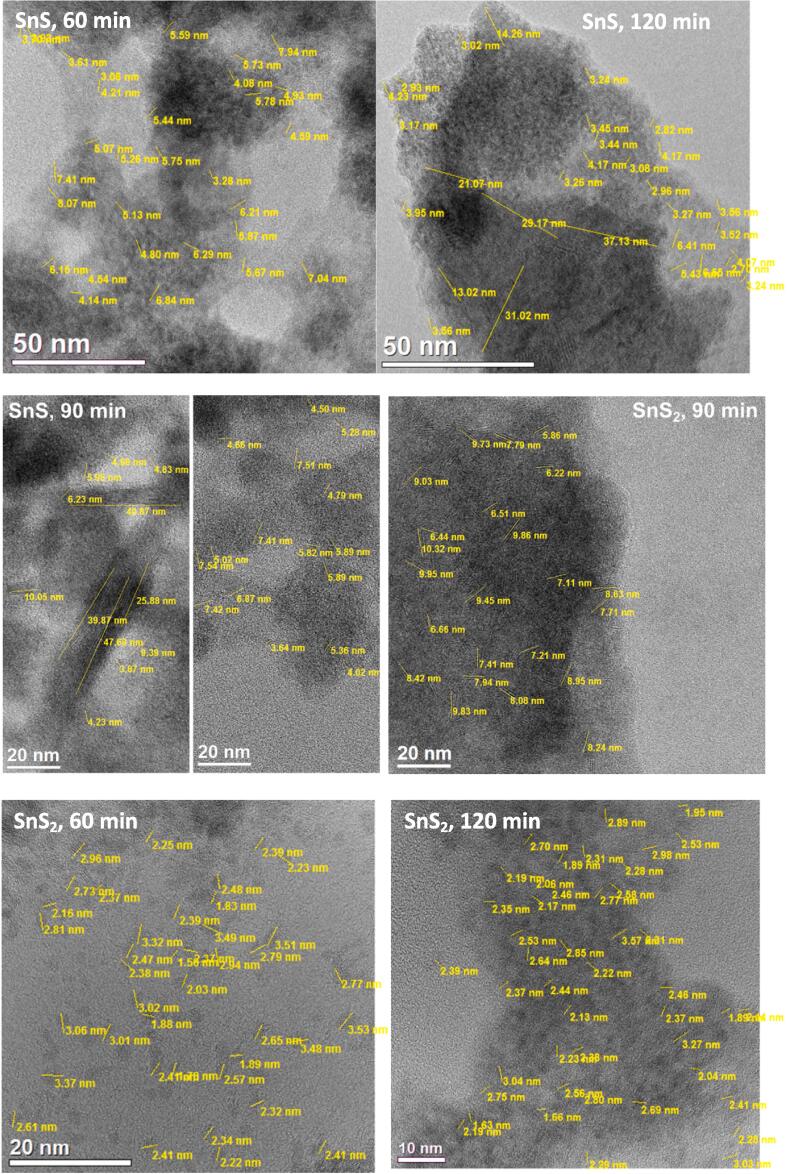
HR-TEM images of sonochemically synthesized SnS and SnS_2_ powders.

The high-resolution transmission electron microscopy observations of SnS samples obtained sonochemically reveal the presence of nanocrystallites of size lying in approximate range 3–8 nm for 60 min of sonication. For SnS powders obtained after 90 min of sonication two types of entities could be observed. Generally they formed either polycrystalline clusters with mostly indistinguishable grain boundaries (the ones that could be distinguished have sizes in range 3.5 – 7.5 nm) or longitudinal nanocrystals being max. 40–50 nm long with width not exceeding 5 nm. In the case of SnS powder synthesized under 120 min of sonication nanoparticles of two kinds were observed: one had sizes in range c.a. 3 – 7 nm and the others had sizes in range c.a. 30–––40 nm. However, in the case of sonochemical synthesis of SnS_2_, the nanoparticles had sizes lying in range c.a. 1.5 – 3.5 nm independently from the duration of synthesis. SnS_2_ sample obtained after 90 min of reaction also appeared to be a highly polycrystalline material and most grain boundaries in this case were also difficult to distinguish. In this case, also larger crystallites were observed (i.e. with sizes in range 5 – 10  nm). Generally, synthesized in this study SnS_2_ nanoparticles were smaller than the SnS nanoparticles (Fig. 5).

**Fig. 6:**
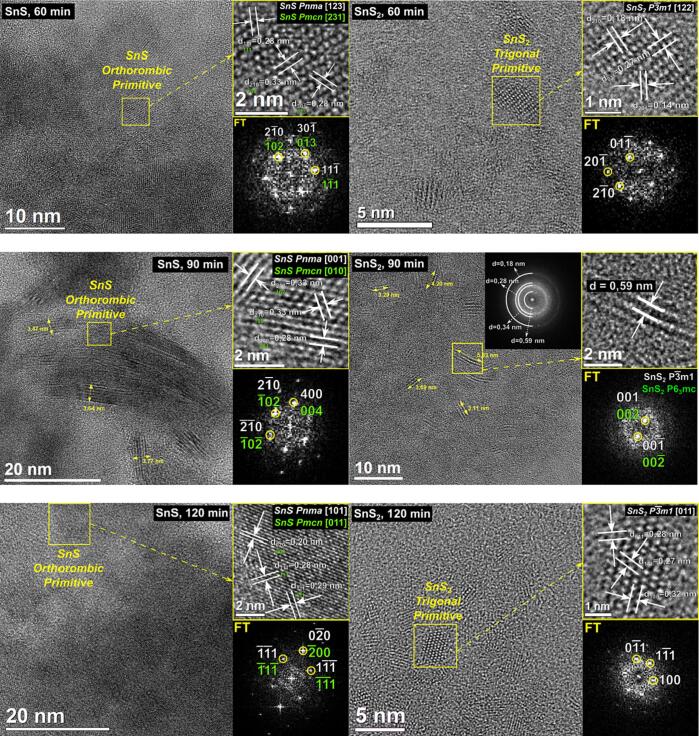
Structural analyses of SnS and SnS_2_ nanoparticles obtained under distinct sonication times, based on HR-TEM images.

Fourier transform analysis of HR-TEM images of SnS particles obtained under sonication lasting 60 min showed that they fit to the orthorombic primitive structure of two possible space groups – *Pnma* of cell parameters a = 11.201(5) Å, b = 3.990(5) Å, c = 4.335(5) Å, α = β = γ = 90° (the crystallite is oriented in [1 2 3] direction) or *Pmcn* of cell parameters a = 3.99(2) Å, b = 4.34(2) Å, c = 11.20(2) Å, α = β = γ = 90° (the crystallite is oriented in [2 3 1] direction) – see Fig. 6. The same kind of agreement was also observed for SnS sample obtained after 90 min of sonication. In this case values of interplanar spacings obtained based on Fourier transform analysis also fitted the same two orthorombic primitive structures of SnS (*Pnma* with crystallite seen in [0 0 1] zone axis and *Pmcn* with crystallite seen in [0 1 0] zone axis). The same was observed for SnS particle in powder obtained under sonication lasting 120 min with the difference that the directions were [1 0 1] and [0 1 1] for *Pnma* and *Pmcn* space groups respectively.

Fig. 6 presents similar structural analyses, based on HR-TEM images, also for SnS_2_ obtained sonochemically. In the case of product of synthesis lasting 60 min analysis of nanoparticle of dimensions c.a. 3.2 x 2.0 nm revealed that it fits a trigonal primitive structure of SnS_2_ with space group *P-3 m1* of cell parameters a = b = 3.638(3) Å, c = 5.88(1) Å, α = β = 90°, γ = 120°. The crystallite is seen in [1 2 2] direction. As mentioned earlier, the SnS_2_ powder obtained after 90 min of reaction is highly polycrystalline and for this sample it was not possible to find single crystallites with a clearly visible atomic arrangement to analyze it in a similar manner as in previous cases. Fourier transform analysis from a whole HR-TEM image allowed to determine four characteristic interplanar spacings which match the spacings of two hexagonal primitive structures (*P-3 m1* (ICSD # 100610) – d_110, 1-20_ = 0.182 nm, d_011_ = 0.278 nm, d_010_ = 0.315 nm and d_001_ = 0.588 nm and *P6_3_mc* (ICSD # 43003) - d_110, 1-20_ = 0.182 nm, d_012, 1-12_ = 0.278 nm, d_010_ = 0.315 nm and d_002_ = 0.591 nm). Interestingly, for this sample single nanocrystals (with sizes in range 3–10 nm, see Fig. 5) with one specific atomic plane could be observed. Its interplanar distance equals 0.59 nm and it's one of the four characteristic distances measured for the polycrystalline material. As mentioned above, this value corresponds to the {0 0 1} planes of the hexagonal primitive structure with *P-3 m1* space group or the {0 0 2} planes of hexagonal primitive structure with *P6_3_mc* space group. In the case of SnS_2_ synthesized under 120 min of sonication a nanoparticle of dimensions c.a. 2.5 x 3.5 nm was analyzed. It also fits a trigonal primitive structure of space group *P-3 m1*. The crystallite is oriented in [0 1 1] direction.

The calculations performed using the Scherrer equation for SnS samples, taking K = 0.9 and.

λ = 0.154056 nm, gave following values of crystallites mean sizes: 4.5 nm (60 min long synthesis), 6.2 nm (90 min long synthesis), and 6.4 nm (120 min long synthesis). These values are in good agreement with the HR-TEM observations. For SnS_2_ samples such calculations weren’t possible due to insufficient quality of powder diffractograms for this samples.

It is postulated that the sulphides are formed via precipitation due to the formation of hydrogen sulfide formed in the hydrolysis of thioacetamide:

CH_3_CSNH_2_ + 2 H_2_O → CH_3_COOH + 2 H_2_S + NH_3_.

SnCl_2_ + H_2_S → SnS + 2 HCl.

SnCl_4_ + 2 H_2_S → SnS_2_ + 4 HCl.

Taking into account that the size of nanocrystallites is rather similar towards different synthesis times, it is postulated that the main mechanism of growth of nanoparticles is the precipitation mechanism and, once formed, the nanoparticles do not expand significantly.

### 3.7 X-ray photoelectron spectroscopy

The concentration of main elements was estimated from high resolution XPS spectra and collected in Table 2. The presence of tin, sulfur as well as carbon and oxygen was found on the surface of the tested samples. At the level of few percentage nitrogen and chlorine were also detected in wide spectra. As they come from technological process they were not considered in analysis.

**Table 2:** Results of the XPS and EDS elemental composition (at %) of tested samples from high resolution spectra.

	XPS					EDS
Sample	Sn	S	C	O	Sn:S ratio	Sn:S ratio
SnS_2_ (60 min)	19.6	20.4	21.6	38.4	0.96	0.91
SnS_2_ (90 min)	23.2	15.3	35.1	26.4	1.52	1.27
SnS_2_ (120 min)	4.6	3.8	68.9	22.7	1.20	0.50
SnS (60 min)	3.5	3.3	77.2	16.0	1.06	1.12
SnS (90 min)	22.5	7.4	35.9	34.2	3.0	7.77
SnS (120 min)	25.0	9.3	37.7	28.0	2.70	1.10

**Fig. 7:**
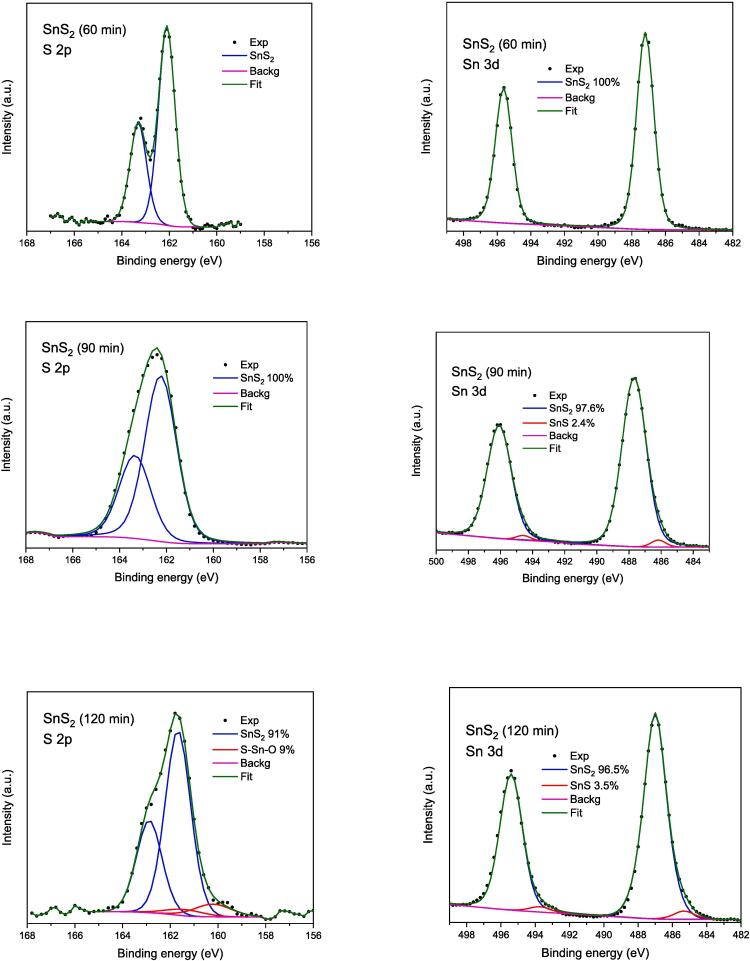
XPS spectra of sonochemically synthesized SnS_2_ quantum dots.

**Fig. 8:**
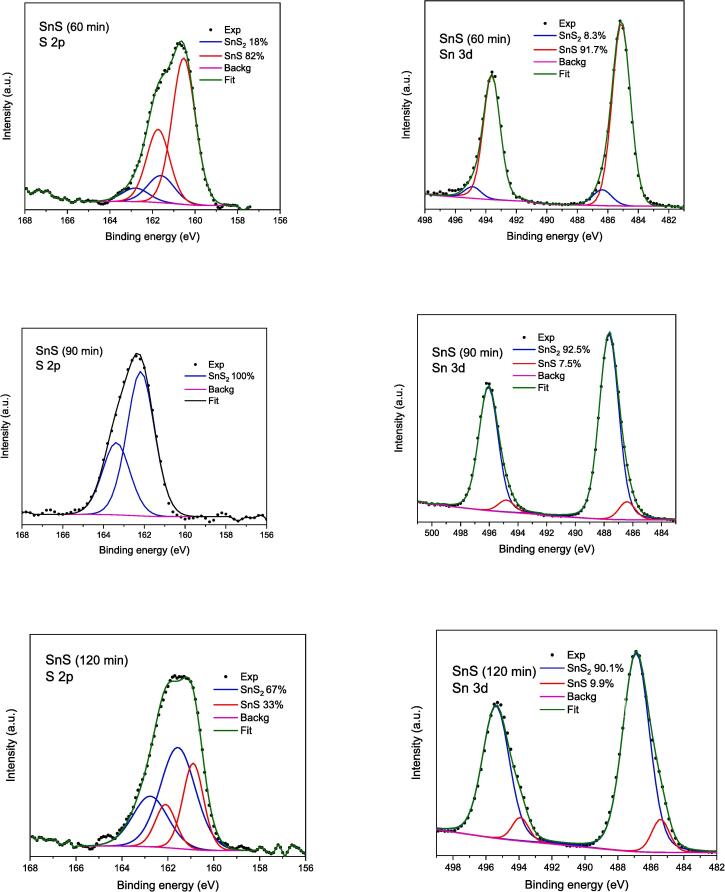
XPS spectra of sonochemically synthesized SnS quantum dots.

The analysis of Sn 3d5/2 and S 2p lines (Fig. 7, Fig. 8 and Table 3) was focused on the evaluation of the chemical states of Sn and S in the studied samples. The XPS survey scans are provided in supplemental materials as Figures S2-S6. In Table 3 the binding energy and the full width at half maximum (FWHM) of given component is collected. The binding energy (BE) of Sn(II) 3d5/2 line in SnS (BE = 486.0 eV) is about 1 eV smaller than Sn(IV) in SnS_2_ (BE = 487.0 eV) [59]. For Sn(0) BE = 484.8 eV was reported [60]. Moreover, the BE of Sn in oxides is very similar to that in sulphides [60]. Therefore, if the content of Sn and S differs from stoichiometry of SnS and SnS_2_, which is observed for both SnS_2_ samples and SnS (120 min) nanoparticles (see Table 2), we can assume that excess of Sn is bonded with oxygen. The chemical shift between S 2p3/2 bonded in SnS (BE = 160.9 eV) and in SnS_2_ (BE = 162.1 eV) is also close to 1 eV. From Table 3 one can see that only for sample SnS_2_ (60 min) one component was formed with quite narrow lines. For all other samples we found two components in different proportions. In sample SnS_2_ (120 min) 9 % of S was bonded in SnS form and in SnS (60 min) still 17.6 % S was in SnS_2_ form. Moreover, for SnS (90 min) all sulphur was in SnS_2_ and for SnS (120 min) most of the sulphur was in SnS_2_ form.

**Table 3:** The binding energy (eV); percentage of given fraction, and full width at half maximum (eV) (FWHM) for chemical components in analysed samples. FWHM indicates the level of chemical order in formed compounds.

Sample	BE of S in SnS FWHM	BE of S in SnS_2_ FWHM	BE of Sn(II) in SnS FWHM	BE of Sn(IV) in SnS_2_ FWHM
SnS_2_ (60 min)	–	161.9;100 % 0.8	–	487.0; 100 % 1.2
SnS_2_ (90 min)	–	161.6; 100 % 1.6	485.5; 2.4 % 1.1	487.0; 97.6 % 1.8
SnS_2_ (120 min)	160.2; 9 % 1.8	161.7; 91 % 1.2	485.8; 3.5 % 1.4	487.0; 96.5 % 1.6
SnS (60 min)	160.5; 82.4 % 1.3	161.6; 17.6 % 1.5	485.7; 91.7 % 1.4	487.0; 8.3 % 1.3
SnS(90 min)	–	161.7; 100 % 1.6	485.9; 7.5 % 1.3	487.2; 92.5 1.6
SnS (120 min)	160.9; 33.4 % 1.1	161.6; 66.6 % 1.8	486.2; 9.9 % 1.9	487.1; 90.1 % 1.3

### 3.8 Determination of energy bandgap value using Tauc method

**Fig. 9:**
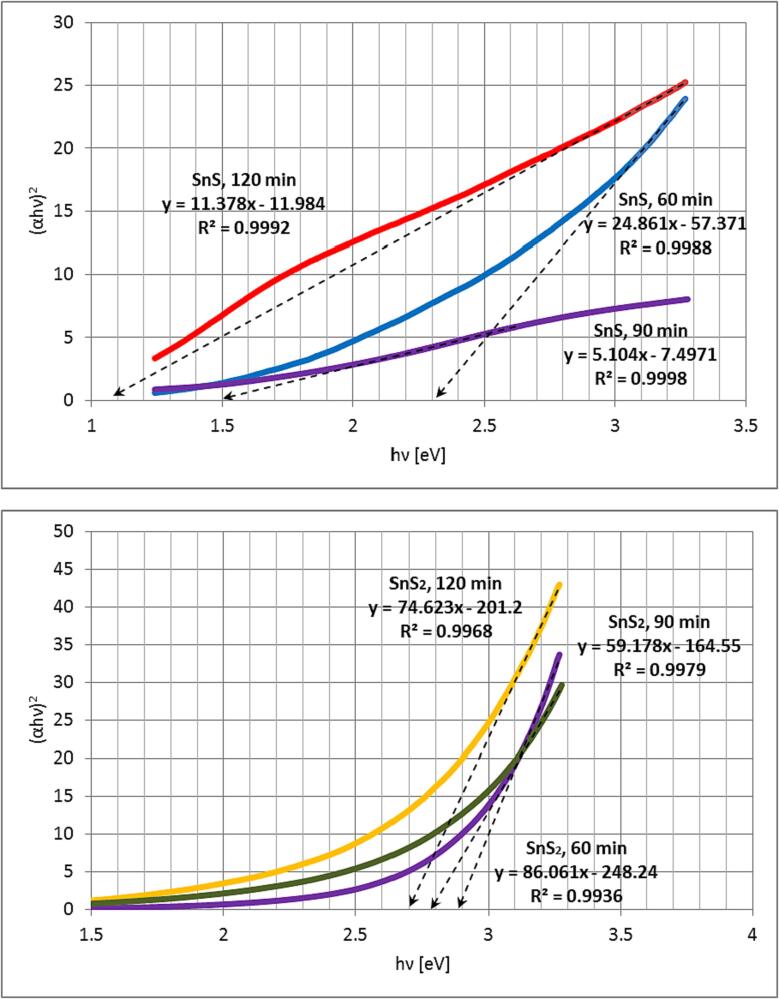
Tauc plots for direct optical transitions for sonochemically obtained tin sulphides.

The results of analyses based on Tauc method show that synthesized samples differ greatly in value of direct optical energy bandgap (Fig. 9). Corresponding absorption spectra are provided in the supplemental materials as Figures S7 and S8. SnS quantum dots have direct energy bandgap of value 2.31 eV, 1.47 eV and 1.05 eV for products of synthesis lasting 60, 90 and 120 min respectively. On the other hand, SnS_2_ quantum dots have direct energy bandgap of value 2.88 eV, 2.78 eV and 2.70 eV for products of synthesis lasting 60, 90 and 120 min respectively. In the case of SnS, the difference between samples is significant, in contrast to the case of samples of SnS_2_. The referential, literature values of bandgaps for bulk SnS and SnS_2_ are 1.32 eV and 2.38 eV respectively [22], [61], [62]. Quantum dots of SnS obtained under sonication lasting 60 min exhibit value of optical bandgap much greater than the literature one (2.31 eV versus 1.32 eV). This is likely caused by the effect of widening of bandgaps in nanomaterials as this sample consists of quantum dots of sizes equal to just a few nanometers. The same is observed for each synthesized nano-SnS_2_ samples (2.88 eV and 2.70 eV versus 2.31 eV), however powder of SnS_2_ obtained under sonication lasting 120 min has slightly lower bandgap (2.70 eV) than the one obtained under sonication lasting 60 min (2.81 eV), despite the fact that both samples consist of nanoparticles of similar sizes. For both compounds the value of energy bandgap of product of synthesis decreases while increasing the sonication time. This relatively slight difference in values of optical bandgaps may be likely caused by the greater amount of SnO_2_ on the surface of SnS_2_ obtained under sonication lasting 60 min, as revealed by the XPS investigation.

### 3.9 Sonocatalytic and photocatalytic activity

**Fig. 10:**
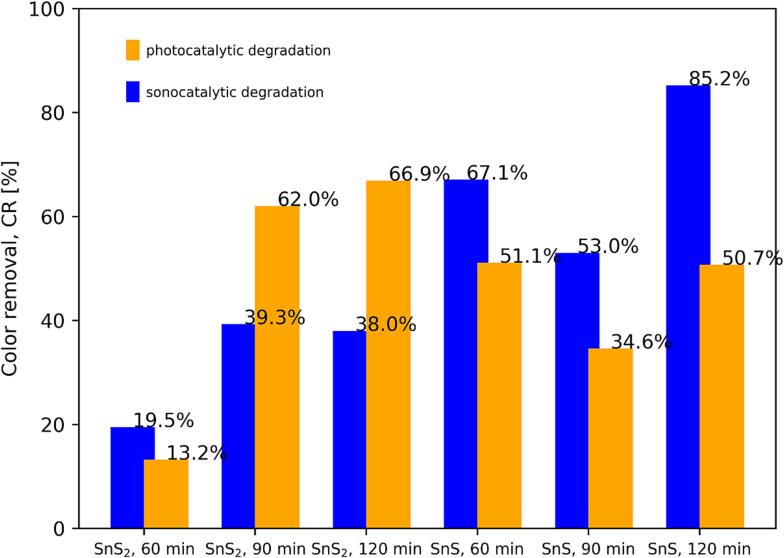
Values of mean percentage color removal for sonochemically synthesized quantum dots of SnS and SnS_2_ under varied sonication time, in two different degradation processes: photo- and sonocatalytic.

Each obtained nanopowder was applied as catalyst in the photo- and sonodegradation of azo-dye Metanil Yellow. The values of mean percentage color removal, CR, for each of investigated tin sulphide quantum dots and for each process were presented on Fig. 10. The greatest color removal was observed in the sonocatalytic degradation applying SnS quantum dots, synthesized through sonication lasting 120 min, as catalyst (85.2 % color removal). Overall, the sonocatalytic process was more efficient than the photocatalytic one. However, exceptions were observed when as catalyst quantum dots of SnS_2_ prepared in synthesis lasting 90 and 120 min were used. In these cases the photocatalytic process showed, respectively, 62.0 % and 66.9 % color removal while in the sonocatalytic process the removal was, respectively, 39.3 % and 38 % only. Quantum dots of SnS_2_ obtained under sonication lasting 120 min were the best photocatalyst, while quantum dots of SnS obtained under similar sonication time were the best sonocatalyst. The worst catalyst, in both sono- and photocatalytic degradation (color removal 19.5 % and 13.2 % respectively), were quantum dots of SnS_2_ obtained in synthesis lasting 60 min. Additionally, it may be seen from Fig. 10 that prepared SnS quantum dots exhibited in both the photo- and sonocatalytic activity for powder prepared under sonication lasting 90 min.

The photocatalytic process depends on the absorption of incident photon by the catalyst thus the activity of catalyst should depend on the value of optical energy bandgap. In this study, the best photocatalyst had bandgap of 2.70 eV value, however there is no explicit dependence between photocatalytic activity and optical bandgap among tested samples. This is clearly shown in the case when SnS quantum dots where applied as photocatalysts – they differed much in value of optical bandgap (2.31 eV and 1.05 eV) but exhibited similar color removal under the same conditions. Despite the fact that for the SnS_2_ samples the longer the synthesis time the lower the energy bandgap, there must be additional factor that affects the photocatalytic activity. On the other hand, the sonocatalytic process consists of three main effects: promotion of acoustic cavitation, photoexcitation by light generated by sonoluminescence, and thermal excitation by heat generated from collapse of acoustic bubbles [44]. Many parameters affect each of these three mechanisms: the value of optical bandgap should affect the photoexcitation, shape and sizes of particles of catalyst should affect the ability for promotion of ultrasonic cavitation and thereby these parameters should also affect the thermal excitation (more acoustic cavitation = more heat generated), also the ability to thermal excitation depends on kind of material of which catalyst is made. The detailed mechanisms of photo- and sonocatalytic processes should be more investigated (in general) to let explain the differences among tested samples of nano tin sulphides. In our study there is no clear correlation between crystallite sizes, optical energy bandgap, and chemical states (Sn(II)/Sn(IV) on the surface of nanoparticles) on the catalytic activity. Probably the observed differences in photo- and sonocatalytic activities is caused by complex interplay of these factors.

## 4 Conclusions

We synthesized quantum dots of SnS and SnS_2_ in sonochemical synthesis using water as a solvent and thioacetamide as sulfur source. We provided values of the sonochemical efficiencies of nano tin sulphides syntheses under distinct sonication times what allows for scaling up of the process. Generally, the syntheses of SnS_2_ quantum dots were more energetically efficient than the syntheses of SnS quantum dots as sonochemical efficiencies for syntheses lasting 120 min were of values 12.98 mg/W and 4.97 mg/W, respectively. Performed characterization of products of syntheses confirmed the desired composition of samples (SnS and SnS_2_) and proved that products of syntheses consists of quantum dots of typical sizes in range 3–8 nm for SnS and 1.5 – 10 nm for SnS_2_. In the case of synthesis of SnS lasting 90 and 120 min also larger nanoparticles, of sizes in range 30–––50 nm, were observed. Raman studies confirmed the structural homogeneity of products of syntheses of which the desired product was tin(IV) sulphide. Each sample had a SnS_2_ structure. On the other hand, products of syntheses of which the desired product was tin(II) sulphide had characteristic modes for SnS nano grains. The peak at 305 cm^−1^ in these samples can be interpreted as the presence of traces of the Sn_2_S_3_ phase. XPS investigation proved that nanoparticles contain tin mainly on desired oxidation state (Sn(II) for SnS and Sn(IV) for SnS_2_). Exception was observed in the case of nano-SnS synthesized under sonication lasting 90 and 120 min, where on the surface of nanoparticles tin is present in both oxidation states with higher amount of Sn(IV). Moreover, substantial part of Sn in this sample is bonded to oxygen. According to the results of analysis performed with the Tauc method, SnS quantum dots have direct energy bandgap of value 2.31 eV (60 min of synthesis), 1.47 eV (90 min of synthesis), and 1.05 eV (120 min of synthesis). On the other hand, SnS_2_ quantum dots have direct energy bandgap of value 2.88 eV (60 min of synthesis), 2.78 eV (90 min of synthesis), and 2.70 eV (120 min of synthesis). These observations are mainly caused by the quantum confinement effect. The comparison of percentage color removal in the photo- and sonocatalytic degradation of azo-dye Metanil Yellow using synthesized quantum dots as catalysts shows that the most efficient sonocatalyst was nano-SnS obtained in sonochemical synthesis lasting 120 min (85.2 % color removal). On the other hand, the most efficient photocatalyst was nano-SnS_2_ obtained applying the same time of synthesis (66.9 % color removal).

## CRediT authorship contribution statement

**Grzegorz Matyszczak:** Conceptualization, Formal analysis, Investigation, Methodology, Supervision, Visualization, Writing – original draft, Writing – review & editing. **Tomasz Plocinski:** Formal analysis, Investigation, Visualization. **Piotr Dluzewski:** Formal analysis, Investigation, Visualization. **Aleksandra Fidler:** Formal analysis, Investigation, Visualization, Writing – original draft. **Cezariusz Jastrzebski:** Formal analysis, Investigation, Visualization, Writing – original draft. **Krystyna Lawniczak-Jablonska:** Formal analysis, Investigation, Visualization, Writing – original draft. **Aleksandra Drzewiecka-Antonik:** Formal analysis, Investigation, Visualization, Writing – original draft. **Anna Wolska:** Investigation, Visualization. **Krzysztof Krawczyk:** Supervision, Writing – review & editing.

## Declaration of competing interest

The authors declare that they have no known competing financial interests or personal relationships that could have appeared to influence the work reported in this paper.

## References

[1] Suslick K.S., Choe S.-B., Cichowlas A.A., Grinstaff M.W. (1991). Sonochemical synthesis of amorphous iron. Nature.

[2] Okitsu K., Cavalieri F. (2018). Sonochemical production of nanomaterials, SpringerBriefs in molecular science: ultrasound and sonochemistry. Springer Nature.

[3] Gedanken A. (2004). Using sonochemistry for the fabrication of nanomaterials. Ultrason. Sonochem..

[4] Liu Y., Xu J., Ni Z., Fang G., Tao W. (2015). One-step sonochemical synthesis route towards kesterite Cu_2_ZnSnS_4_ nanoparticles. J. Alloys Compd..

[5] Manivannan R., Sahu D., Selvaraju N., Noyelvictoria S. (2019). Single step sonochemical synthesis of copper zinc tin sulfide nanoparticles. J Sci Ind Res India.

[6] Raju N.P., Tripathi D., Lahiri S., Thangavel R. (2023). Heat reflux sonochemical synthesis of Cu_3_BiS_3_ quantum dots: experimental and first-principles investigation of spin-orbit coupling on structural, electronic, and optical properties. Sol Energy.

[7] Pejova B., Sherif E., Minde M.W. (2020). Sonochemically synthesized quantum nanocrystals of cubic CuInS_2_: evidence for multifractal surface morphology, size-dependent structure, and particle size distribution. J. Phys. Chem. C.

[8] Metters J.P., Banks C.E., Pollet B.G., Manickam S., Ashokkumar M. (2014). Cavitation: A Novel Energy-Efficient Technique for the Generation of Nanomaterials.

[9] Pollet B.G. (2010). The use of ultrasound for the fabrication of fuel cell materials. Int J Hydrogen Energy.

[10] Islam M.H., Paul M.T.Y., Burheim O.S., Pollet B.G. (2019). Recent developments in the sonoelectrochemical synthesis of nanomaterials. Ultrason. Sonochem..

[11] Ahmadi S., Mesbah M., Igwegbe C.A., Ezeliora C.D., Osagie C., Khan N.A., Dotto G.L., Salari M., Dehghani M.H. (2021). Sono electro-chemical synthesis of LaFeO_3_ nanoparticles for the removal of fluoride: optimization and modeling using RSM, ANN and GA tools. J. Environ. Chem. Eng..

[12] Lewis D.J., Kevin P., Bakr O., Muryn C.A., Malik M.A., O’Brien P. (2014). Routes to tin chalcogenide materials as thin films or nanoparticles: a potentially important class of semiconductor for sustainable solar energy conversion. Inorg. Chem. Front..

[13] Reddy N.K., Devika M., Gopal E.S.R. (2015). Review on tin (II) sulfide (SnS) material: synthesis, properties, and applications. Crit. Rev. Solid State.

[14] Zhang Q.X., Ma S.Y., Zhang R., Tie Y., Pei S.T. (2020). Optimization ethanol detection performance manifested by SnS/SnS_2_ nanoparticles. Mater. Lett..

[15] Wu Y.-Q., Yang Y., Pu H., Gao R.-Z., Meng W.-J., Yang H.-X., Zhao D.-L. (2020). SnS_2_ nanoparticle-integrated graphene nanosheets as high-performance and cycle-stable anodes for lithium and sodium storage. J. Alloys Compd..

[16] Tian H., Fan C.h., Liu G., Yuan S.h., Zhang Y., Wang M., Li E. (2019). Ultrafast broadband photodetector based on SnS synthesized by hydrothermal method. Appl. Surf. Sci..

[17] Zhu H., Yang D., Ji Y., Zhang H., Shen X. (2005). Two-dimensional SnS nanosheets fabricated by a novel hydrothermal method. J. Mater. Sci..

[18] An C., Tang K., Shen G., Wang C., Yang Q., Hai B., Qian Y. (2002). Growth of belt-like SnS crystals from ethylenediamine solution. J. Cryst. Growth.

[19] Hickey S.G., Waurisch C., Rellinghaus B., Eychmüller A. (2008). Size and shape control of colloidally synthesized IV-VI nanoparticulate TIN(II) sulfide. J. Am. Chem. Soc..

[20] Liu Y., Hou D., Wang G. (2003). Synthesis and characterization of SnS nanowires in cetyltrimethylammoniumbromide (CTAB) aqueous solution. Chem. Phys. Lett..

[21] Shen G., Chen D., Tang K., Huang L., Qian Y., Zhou G. (2003). Novel polyol route to nanoscale tin sulfides flaky crystallines. Inorg. Chem. Commun..

[22] Gajendiran J., Rajendran V. (2011). Synthesis of SnS_2_ nanoparticles by a surfactant-mediated hydrothermal method and their characterization. Adv. Nat. Sci.: Nanosci. Nanotechnol..

[23] Xiao H., Zhang Y.C. (2008). In air synthesis of SnS_2_ nanoplates from tin, sulfur and ammonium choride powders. Mater. Chem. Phys..

[24] Giberti A., Gaiardo A., Fabbri B., Gherardi S., Guidi V., Malagu C., Bellutti P., Zonta G., Casotti D., Cruciani G. (2016). TIN(IV) sulfide nanorods as a new gas sensing material. Sens Actuators B Chem.

[25] Mukaibo H., Yoshizawa A., Momma T., Osaka T. (2003). Particle size and performance of SnS_2_ anodes for rechargeable lithium batteries. J. Power Sources.

[26] Cheraghizade M., Jamali-Sheini F., Yousefi R., Niknia F., Mahmoudian M.R., Sookhakian M. (2017). The effect of tin sulfide quantum dots size on photocatalytic and photovoltaic performance. Mater. Chem. Phys..

[27] Khimani A.J., Chaki S.H., Chauhan S.M., Mangrola A.V., Meena R.R., Deshpande M.P. (2019). Synthesis, characterization, antimicrobial and antioxidant study of the facile sonochemically synthesized SnS_2_ nanoparticles. Nano-Struct. Nano-Objects..

[28] Jamali-Sheini F., Yousefi R., Bakr N.A., Cheraghizade M., Sookhakian M., Huang N.M. (2015). Highly efficient photo-degradation of methyl blue and band gap shift of SnS nanoparticles under different sonication frequencies. Mat. Sci. Semicon. Proc..

[29] Malikov E.Y. (2020). Potential semiconductor material based on the multiwall carbon nanotube – maleic anhydride – 1-octene/SnS nanocomposite, compos. Interfaces.

[30] Matyszczak G., Fidler A., Polesiak E., Sobieska M., Morawiec K., Zajkowska W., Lawniczak-Jablonska K., Kuzmiuk P. (2020). Application of sonochemically synthesized SnS and SnS_2_ in the electro-Fenton proces: kinetics and enhanced decolorization. Ultrason. Sonochem..

[31] Matyszczak G., Jóźwik P., Polesiak E., Sobieska M., Krawczyk K., Jastrzębski C., Płociński T. (2021). Sonochemical preparation of SnS and SnS_2_ nano- and micropowders and their characterization. Ultrason. Sonochem..

[32] Gao Y., Bai L., Zhang X., Yang F. (2021). Non-parallel photo-assisted electrocatalysis mechanism of SnS_2_/NiO heterojunction for efficient electrocatalytic oxygen evolution reaction. ChemElectroChem.

[33] Huaizhang W., Huaning J., Ting L. (2021). E3S Web of Conferences 267.

[34] Liang A., Ming J., Zhu W., Guan H., Han X., Zhang S., Lin Y., Dong J., Huang Y., Qiu W., Lu H., Zheng H., Zhang Y., Yu J., Chen Z., Peng G. (2021). Tin disulfide-coated microfiber for humidity sensing with fast response and high sensitivity. Crystals.

[35] Liu X., Najam T., Yasin G., Kumar M., Wang M. (2021). One-pot synthesis of high-performance tin Chalcogenides/C anodes for li-ion batteries. ACS Omega.

[36] Zhu C., Wan F., Ping H., Wang H., Wang W., Fu Z. (2021). Biotemplating synthesis of rod-shaped tin sulfides assembled by in-terconnected nanosheets for energy storage. J. Power Sources.

[37] Qiao L., Yu C., Sun R., Tao Y., Li Y., Yan Y. (2021). Three-dimensional magnetic stannic disulfide composites for the solid-phase extraction of sulfonamide antibiotics. J. Chromatogr. A.

[38] Gao F., Chen H., Feng W., Hu Y., Shang H., Xu B., Zhang J., Xu C.-Y., Hu P. (2021). High-performance van der waals metal-insulator-semiconductor photodetector optimized with valence band matching. Adv. Funct. Mater..

[39] Arunkumar M., Veerakumar S., Mohanavel V., Vairamuthu J., Vijayan V., Senthilkumar N. (2021). A novel visible light-driven p-type BiFeO_3_/n-type SnS_2_ heterojunction photocatalyst for efficient charge separation and enhanced photocatalytic activity. J Clust Sci.

[40] Sharma B.M., Becanova J., Scheringer M., Sharma A., Bharat G.K., Whitehead P.G., Klanova J., Nizzetto L. (2019). Health and ecological risk assessment of emerging contaminants (pharmaceuticals, personal care products, and artificial sweeteners) in surface and groundwater (drinking water) in the Ganges River basin, India. Sci. Total Environ..

[41] Lapworth D.J., Das P., Shaw A., Mukherjee A., Civil W., Petersen J.O., Gooddy D.C., Wakefield O., Finlayson A., Krishan G., Sengupta P., MacDonald A.M. (2018). Deep urban groundwater vulnerability in India revealed through the use of emerging organic contaminants and residence time tracers. Environ. Pollut..

[42] Köck-Schulmeyer M., Ginebreda A., Postigo C., Garrido T., Fraile J., López de Alda M., Barceló D. (2014). Four-year advanced monitoring program of polar pesticides in groundwater of Catalonia (NE-Spain). Sci. Total Environ..

[43] Sangamnere R., Misra T., Bherwani H., Kapley A., Kumar R. (2023). A critical review of conventional and emerging wastewater treatment technologies. Sustain. Water Resour. Manag..

[44] Qiu P., Park B., Choi J., Thokchom B., Pandit A.B., Khim J. (2018). A review on heterogeneous sonocatalyst for treatment of organic pollutants in aqueous phase based on catalytic mechanism. Ultrason. Sonochem..

[45] Tizhoosh N.Y., Khataee A., Hassandoost R., Soltani R.D.C., Doustkhah E. (2020). Ultrasound-engineered synthesis of WS_2_@CeO_2_ heterostructure for sonocatalytic degradation of tylosin. Ultrason. Sonochem..

[46] Wang J., Lv Y., Zhang L., Liu B., Jiang R., Han G., Xu R., Zhang X. (2010). Sonocatalytic degradation of organic dyes and comparison of catalytic activities of CeO_2_/TiO_2_, SnO_2_/TiO_2_ and ZrO_2_/TiO_2_ composites under ultrasonic irradiation. Ultrason. Sonochem..

[47] Casa Software Ltd, (n.d.) http://www.casaxps.com/.

[48] Nath P.P., Sarkar K., Tarafder P., Mondal M., Das K., Paul G. (2015). Practice of using metanil yellow as food colour to process food in unorganized sector of West Bengal – a case study. Int. Food Res. J..

[49] Arabkhani S., Pourmoslemi S.h., Harchegani A.L. (2022). Rapid determination of metanil yellow in turmeric using a molecularly imprinted polimer dispersive solid-phase extraction and visible light spectrophotometry. Food Chem..

[50] Levine W.G. (1991). Metabolism of azo dyes: implication for detoxication and activation. Drug Metab Rev..

[51] Chung K.T. (2016). Azo dyes and human health: a review. J Environ Sci Health C Environ Carcinog Ecotoxicol Rev..

[52] Nagaraja T.N., Desiraju T. (1993). Effects of chronic consumption of metanil yellow by developing and adult rats on brain regional levels of noradrenaline, dopamine and serotonin, on acetylcholine esterase activity and on operant conditioning. Food Chem Toxicol..

[53] Sohila S., Rajalakshmi M., Chanchal Ghosh A.K., Arora C.M. (2011). Optical and raman scattering studies on SnS nanoparticles. J. Alloys Compd..

[54] Bialoglowski M., Jastrzebski C., Podsiadlo S., Jastrzebski D.J., Gajda R., Gebicki W., Wrzosek P.A., Wozniak K. (2015). Synthesis of tin disulfide single crystals for nano-layer exfoliation. Cryst. Res. Technol..

[55] Huang Y., Sutter E., Sadowski J.T., Cotlet M., Monti O.L.A., Racke D.A., Neupane M.R., Wickramaratne D., Lake R.K., Parkinson B.A., Sutter P. (2014). Tin disulfide-an emerging layered metal dichalcogenide semiconductor: materials properties and device characteristics. ACS Nano.

[56] Sunny A., Thirumurugan A., Balasubramanian K. (2020). Laser induced Fano scattering, electron-phonon coupling, bond length and phonon lifetime changes in α-Fe2O3 nanostructures. Phys. Chem. Chem. Phys..

[57] Gao Y., Yin P. (2017). Origin of asymmetric broadening of raman peak profiles in si nanocrystals. Sci. Rep..

[58] Chaki S.H., Chaudhary M.D., Deshpande M.P. (2014). Synthesis and characterization of different morphological SnS nanomaterials. Adv. Nat. Sci.: Nanosci. Nanotechnol..

[59] Chia X., Lazar P., Sofer Z., Luxa J., Pumera M. (2016). Layered SnS versus SnS_2_: valence and structural implications on electrochemistry and clean energy electrocatalysis. J. Phys. Chem. C.

[60] Crist B.V. (1999). Handbooks of monochromatic XPS spectra. XPS International LLC.

[61] Raadik T., Grossberg M., Raudoja J., Traksmaa R., Krustok J. (2013). Temperature-dependent photoreflectance of SnS crystals. J. Phys. Chem. Solids.

[62] Burton L.A., Whittles T.J., Hesp D., Linhart W.M., Skelton J.M., Hou B., Webster R.F., O’Dowd G., Reece C., Cherns D., Fermin D.J., Veal T.D., Dhanak V.R., Walsh A. (2016). Electronic and optical properties of single crystal SnS_2_: an earth-abundant disulfide photocatalyst. J. Mater. Chem. A.

