# Supplementary material for: Sonochemical synthesis of SnS and SnS2 quantum dots from aqueous solutions, and their photo- and sonocatalytic activity
Source: Ultrason Sonochem. 2024 Mar 6;105:106834. doi: 10.1016/j.ultsonch.2024.106834 (PMC10981103; doi:10.1016/j.ultsonch.2024.106834)
Supplement: Supplementary data 1 [file mmc1.docx]

*Supporting information for:*

Sonochemical synthesis of SnS and SnS_2_ quantum dots from aqueous solutions, and their photo- and sonocatalytic activity

Grzegorz Matyszczak^a)^*, Tomasz Plocinski^b)^, Piotr Dluzewski^c)^, Aleksandra Fidler^c)^, Cezariusz Jastrzebski^d)^, Krystyna Lawniczak-Jablonska^c)^, Aleksandra Drzewiecka-Antonik^c)^, Anna Wolska^c)^, Krzysztof Krawczyk^a)^

a) Department of Chemical Technology, Faculty of Chemistry, Warsaw University of Technology, Noakowski street 3, 00-664 Warsaw

b) Faculty of Materials Science and Engineering, Warsaw University of Technology, Wołoska street 141A, 02-507 Warsaw

c) Institute of Physics Polish Academy of Sciences, Poland, Lotników avenue 32/46, 02-668 Warsaw

d) Faculty of Physics, Warsaw University of Technology, Koszykowa street 75, 00-662 Warsaw

Corresponding author: Grzegorz Matyszczak, [grzegorz.matyszczak@pw.edu.pl](mailto:grzegorz.matyszczak@pw.edu.pl)


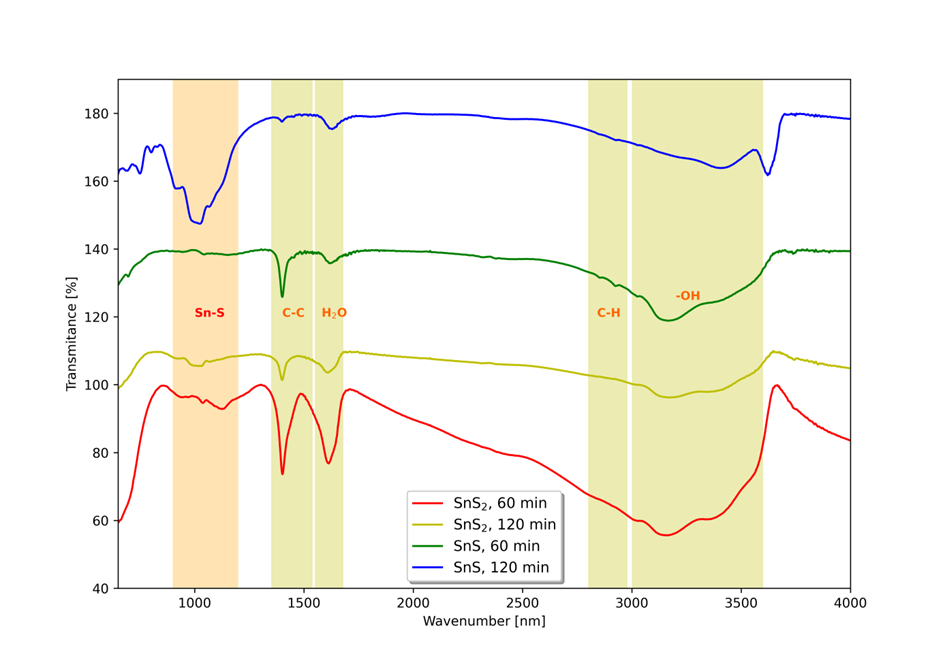


**Figure S1.** FT-IR spectra of synthesized SnS and SnS_2_ samples.

**Figure S2.** XPS survey analysis of SnS_2_ obtained under 60 minutes of sonication.

**Figure S3.** XPS survey analysis of SnS_2_ obtained under 90 minutes of sonication.


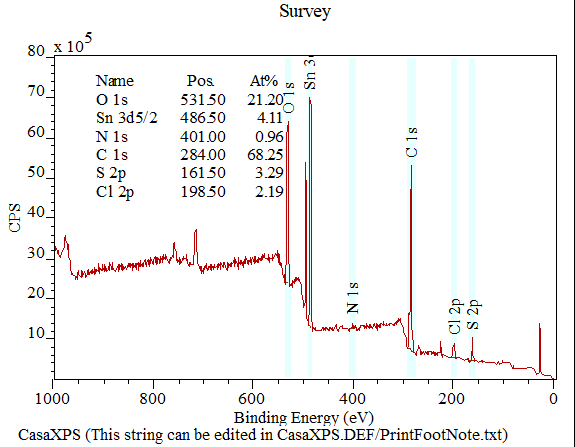


**Figure S4.** XPS survey analysis of SnS_2_ obtained under 120 minutes of sonication.

**Figure S5.** XPS survey analysis of SnS obtained under 90 minutes of sonication.


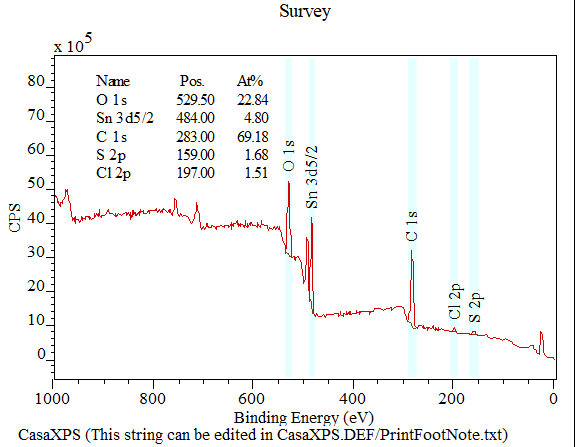


**Figure S6.** XPS survey analysis of SnS obtained under 120 minutes of sonication.


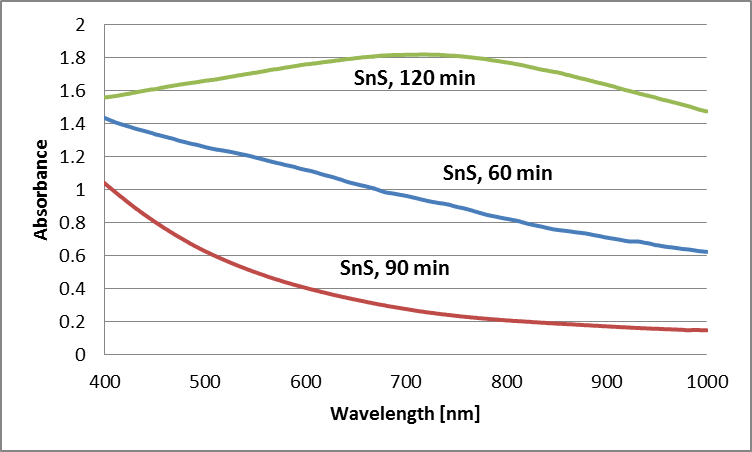


**Figure S7.** Absorption spectra of SnS samples.


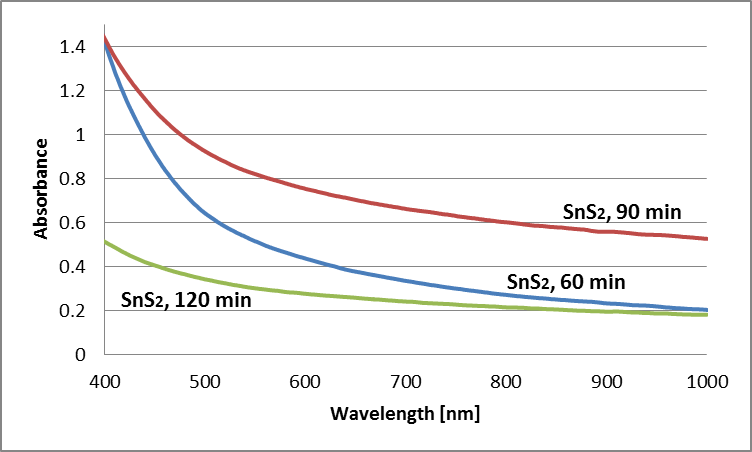


**Figure S8.** Absorption spectra of SnS_2_ samples.
